# Oil and Gas Projects in the Western Amazon: Threats to Wilderness, Biodiversity, and Indigenous Peoples

**DOI:** 10.1371/journal.pone.0002932

**Published:** 2008-08-13

**Authors:** Matt Finer, Clinton N. Jenkins, Stuart L. Pimm, Brian Keane, Carl Ross

**Affiliations:** 1 Save America's Forests, Washington D. C., United States of America; 2 Nicholas School of the Environment, Duke University, Durham, North Carolina, United States of America; 3 Land Is Life, Somerville, Massachusetts, United States of America; Stanford University, United States of America

## Abstract

**Background:**

The western Amazon is the most biologically rich part of the Amazon basin and is home to a great diversity of indigenous ethnic groups, including some of the world's last uncontacted peoples living in voluntary isolation. Unlike the eastern Brazilian Amazon, it is still a largely intact ecosystem. Underlying this landscape are large reserves of oil and gas, many yet untapped. The growing global demand is leading to unprecedented exploration and development in the region.

**Methodology/Principal Findings:**

We synthesized information from government sources to quantify the status of oil development in the western Amazon. National governments delimit specific geographic areas or “blocks” that are zoned for hydrocarbon activities, which they may lease to state and multinational energy companies for exploration and production. About 180 oil and gas blocks now cover ∼688,000 km^2^ of the western Amazon. These blocks overlap the most species-rich part of the Amazon. We also found that many of the blocks overlap indigenous territories, both titled lands and areas utilized by peoples in voluntary isolation. In Ecuador and Peru, oil and gas blocks now cover more than two-thirds of the Amazon. In Bolivia and western Brazil, major exploration activities are set to increase rapidly.

**Conclusions/Significance:**

Without improved policies, the increasing scope and magnitude of planned extraction means that environmental and social impacts are likely to intensify. We review the most pressing oil- and gas-related conservation policy issues confronting the region. These include the need for regional Strategic Environmental Impact Assessments and the adoption of roadless extraction techniques. We also consider the conflicts where the blocks overlap indigenous peoples' territories.

## Introduction

**Figure 1:**
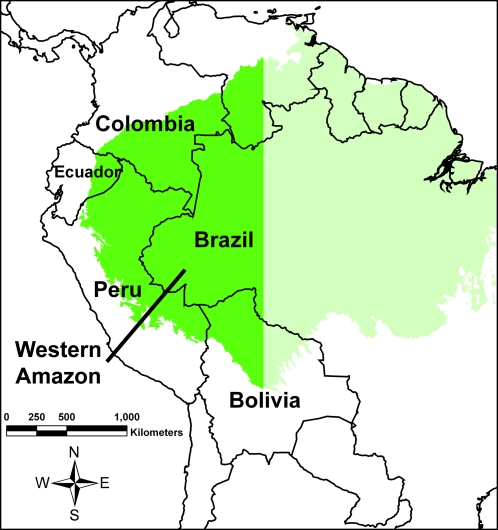
Study area of the western Amazon.

The western Amazon includes parts of Bolivia, Colombia, Ecuador, Peru, and western Brazil (Figure 1). It is one of the most biodiverse areas of the planet for many taxa, including plants, insects, amphibians, birds, and mammals [1]–[7]. The region maintains large tracts of intact tropical moist forest and has a high probability of stable climatic conditions in the face of global warming [8]. By contrast, the eastern Amazon in Brazil, where much of the global attention has focused, has a high probability of continued massive deforestation [9] and drought risk in the coming decades [10]. The western Amazon is also the home to many indigenous ethnic groups, including some of the world's last uncontacted peoples living in voluntary isolation [11]–[13].

Underlying this landscape of extraordinary biological and cultural diversity are large reserves of oil and gas, many yet untapped. Record oil prices and growing global demand are now stimulating unprecedented levels of new oil and gas exploration and extraction. It is the nations of the region, and not the indigenous peoples who live on much of the land, who assert their constitutional ownership of subsoil natural resources. National governments delimit specific geographic areas or “blocks” that are zoned for hydrocarbon activities, which they may lease to state and multinational energy companies for exploration and production.

Oil exploration in the western Amazon started as early as the 1920s in Peru [14] and Ecuador [15], with a production boom arriving in the 1970s. The subsequent three decades have seen numerous large projects, such as several oil projects in the central Ecuadorian Amazon, the Urucu gas project in Brazil, and the Camisea gas project in Peru.

Oil and gas development in the western Amazon has already caused major environmental and social impacts 16–19. Direct impacts include deforestation for access roads, drilling platforms, and pipelines, and contamination from oil spills and wastewater discharges. The technologies of the 1970s-era oil operations caused widespread contamination in the northern Ecuadorian [20]–[21] and northern Peruvian Amazon [22]–[23]. Even the much newer Camisea pipeline, which began operations in the fall of 2004, had five major spills in its first 18 months of operation [24]. A 1990s-era oil operation experienced a major spill in Ecuador's Yasuní region as recently as January 2008 [25]. There are also direct impacts associated with seismic testing activities during the exploration phase of projects [17], [26].

Indirect effects arise from the easy access to previously remote primary forest provided by new oil roads and pipeline routes, causing increased logging, hunting, and deforestation from human settlement [27]–[29]. For example, much of the extensive deforestation in the northern and central Ecuadorian Amazon followed colonization along the oil access roads [30]–[32].

Social impacts are also considerable. The national representative organizations of indigenous peoples in Ecuador (CONAIE) and the Peruvian Amazon (AIDESEP) have opposed new oil and gas projects, citing the widespread contamination from previous and current oil projects [33]–[34]. In both countries, local residents and indigenous peoples have taken legal actions against U.S. oil companies for allegedly dumping billions of gallons of toxic waste into the forests [35]–[37]. Intense opposition from indigenous peoples has stopped exploration in two leased blocks in Ecuador (Blocks 23 and 24) for over seven years [38]. Deforestation and colonization following road building has affected the core territory of several indigenous groups in Ecuador. Oil and gas projects in the territories of indigenous peoples in voluntary isolation have become highly contentious. These peoples, so named due to their decision of avoiding contact with the outside world [11], inhabit remote parts of the western Amazon [11]–[13] and are extremely vulnerable because they lack resistance or immunity from outsiders' diseases [39]. First contact results in high rates of morbidity and mortality, with mortality estimates ranging between a third and half of the population within the first several years [11].

The extent and intensity of oil and gas exploration and development in the western Amazon may soon increase rapidly. Information on the future of oil and gas activities across the entire region is limited. Here, we quantify and map the extent of current and proposed oil and gas activity across the western Amazon using information from government and news sources. We document how the oil and gas blocks overlap areas of peak biodiversity, protected areas, and indigenous territories. Finally, we discuss policy options that might mitigate the impacts.

## Results

**Figure 2:**
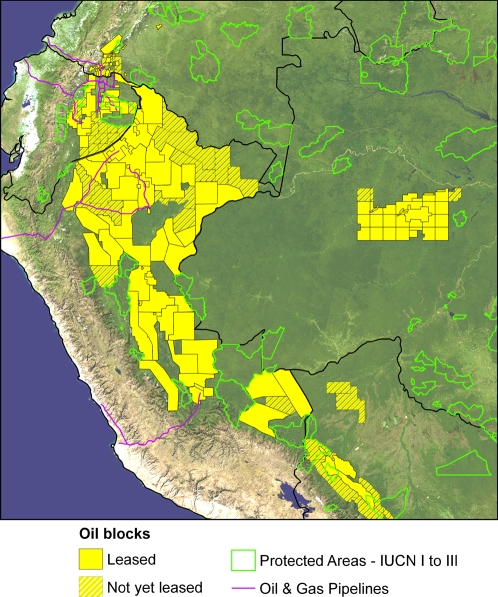
Oil and gas blocks in the western Amazon. Solid yellow indicates blocks already leased out to companies. Hashed yellow indicates proposed blocks or blocks still in the negotiation phase. Protected areas shown are those considered strictly protected by the IUCN (categories I to III).

**Figure 3:**
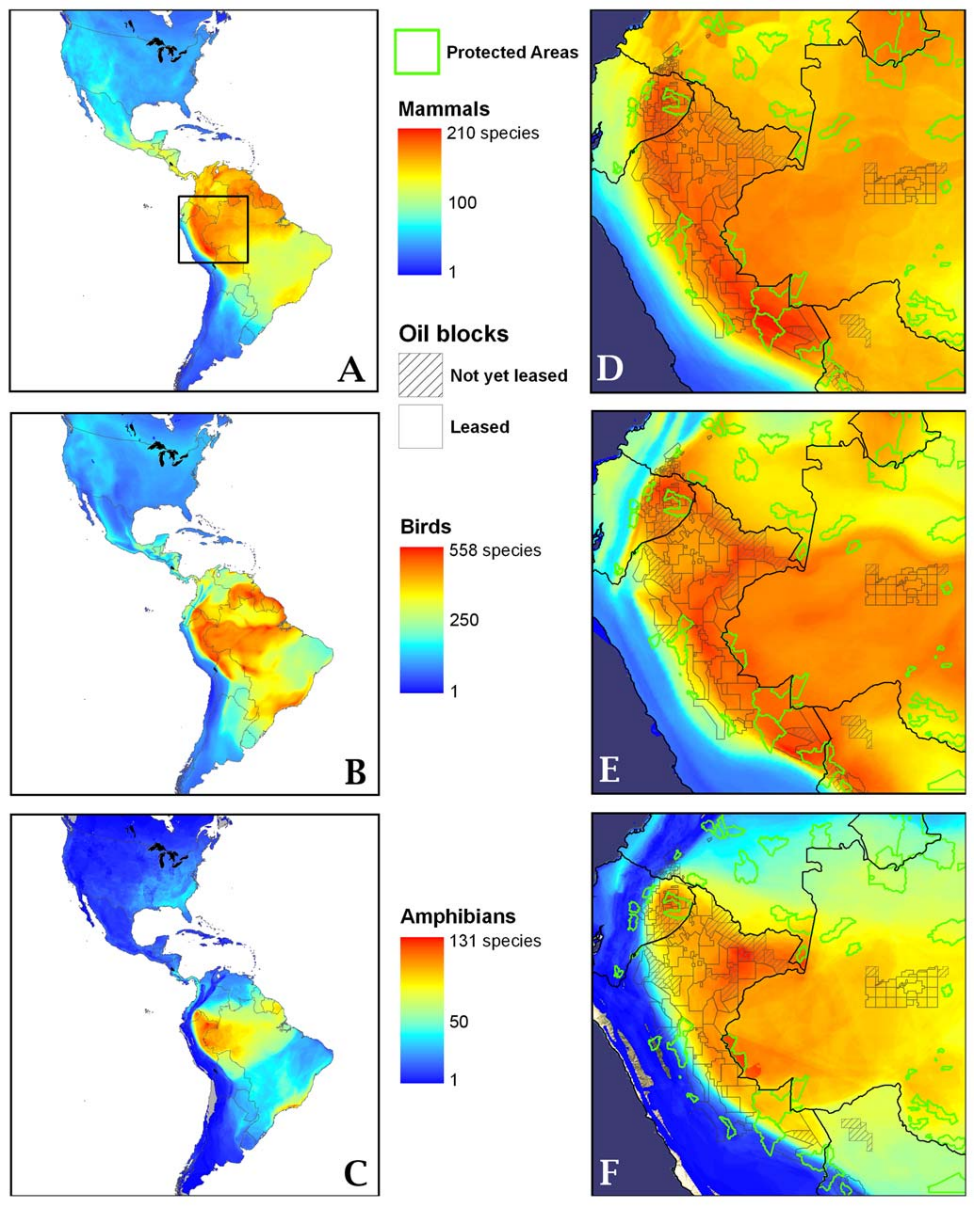
Overlap of oil and gas blocks with biodiversity and protected areas. The number of species of mammals (A), birds (B), and amphibians (C) across the Americas, where the highest diversity occurs in the western Amazon. Detailed view of the western Amazon region, outlined by the box in A, for mammals (D), birds (E), and amphibians (F). In this region hydrocarbon blocks overlap areas of exceptionally high biodiversity. Protected areas shown are those considered strictly protected by the IUCN (categories I to III).

There are now ∼180 oil and gas blocks covering ∼688,000 km^2^ of forest in the western Amazon (Figure 2). At least 35 multinational oil and gas companies operate these blocks, which overlap the most species-rich part of the Amazon for amphibians, birds, and mammals (Figure 3). Oil and gas projects affect the forest of all western Amazonian nations, but to varying degrees. For example, in both Ecuador and Peru blocks now cover more than two-thirds of the Amazon, while in Colombia that fraction is less than one-tenth. In Bolivia and western Brazil, historical impacts are minimal, but the area open to oil and gas exploration is increasing rapidly.

**Figure 4:**
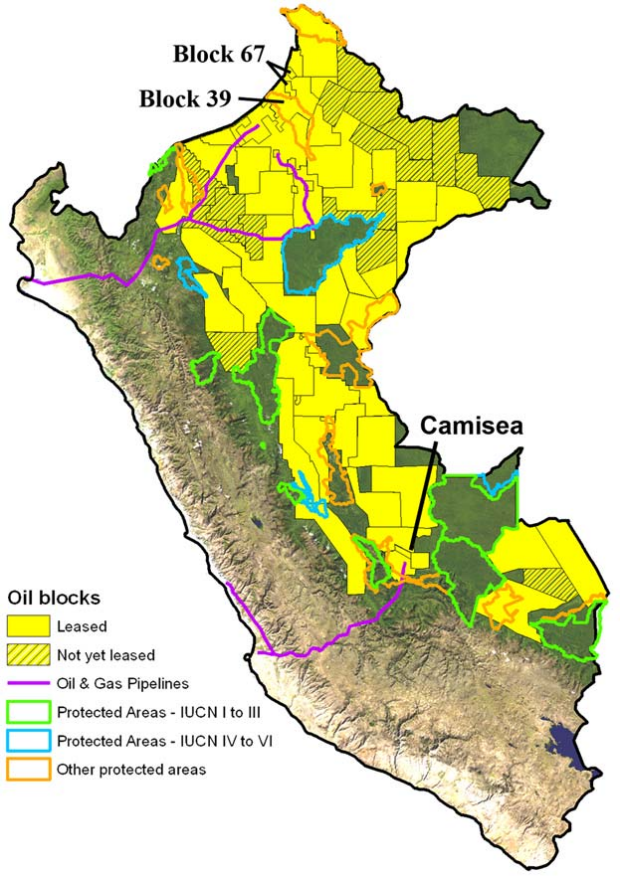
Focus on Peru. Oil and gas blocks in Peru, including all IUCN categorized Amazonian protected areas, protected areas not yet placed in an IUCN category, and key features discussed in the text.

In 2003, Peru reduced royalties to promote investment, sparking a new exploration boom. There are now 48 active blocks under contract with multinational companies in the Peruvian Amazon (Figure 4). The government has leased all but eight in just the past four years. At least 16 more blocks are likely to be signed in 2008. These 64 blocks cover ∼72% of the Peruvian Amazon (∼490,000 km^2^). The only areas fully protected from oil and gas activities are national parks and national and historic sanctuaries, which cover ∼12% of the total Peruvian Amazon. However, 20 blocks overlap 11 less strictly protected areas, such as Communal Reserves and Reserved Zones. At least 58 of the 64 blocks overlay lands titled to indigenous peoples. Further, 17 blocks overlap areas that have proposed or created reserves for indigenous groups in voluntary isolation.

Several large recent oil discoveries in the remote forests on the Peruvian side of the Peru-Ecuador border will likely trigger a new wave of development. Initial estimates indicate over 500 million barrels in Blocks 67 and 39 (labeled in Figure 4), the former of which has recently begun its development phase [40]. Gas development in the Camisea region is likely to continue as well. A new gas discovery in the region announced in January 2008 brought the proven reserves of the Camisea area to over 15 trillion cubic feet. In addition, a wave of exploration is about to begin as the 40 blocks leased out over the last four years begin operations on the ground. In 2007 alone, the government approved the Environmental Impact Studies (EIS, see below) for 10 blocks that are set to begin immediate seismic testing and drilling of exploratory wells.

**Figure 5:**
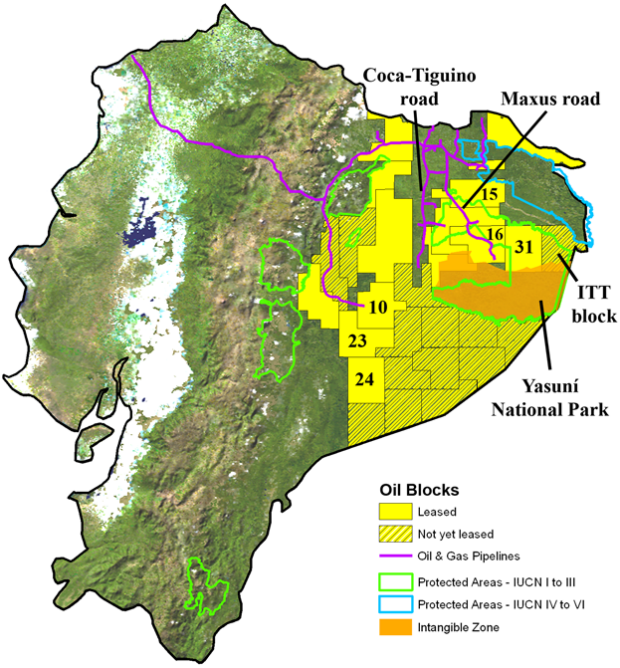
Focus on Ecuador. Oil and gas blocks in Ecuador, including all IUCN categorized Amazonian protected areas and key features discussed in the text. Oil blocks discussed in the text are numbered.

The Ecuadorian government has zoned ∼65% of the Amazon for oil activities (∼52,300 km^2^) (Figure 5). Blocks overlap the ancestral or titled lands of ten indigenous groups. Oil development began in the north in the 1970s. The oil frontier in Ecuador has now shifted south, where a quarter of Ecuador's untapped oil reserves lie in Yasuní National Park, the country's principal Amazonian national park. Unlike Peru, Ecuador permits oil and gas extraction in national parks. In January 2007, the Ecuadorian government, however, delimited a 7,580 km^2^ “Zona Intangible” — an area off-limits to oil, gas, and logging activities — via Presidential Decree in the southern part of Yasuní. It protects a portion of the territory of the Tagaeri and Taromenane, the country's two known indigenous groups in voluntary isolation. To the southwest of Yasuní, intense opposition [38] from indigenous peoples has stopped exploration in two leased blocks (Blocks 23 and 24) for over seven years. Just to the east of these two blocks, the entire southeastern part of the Ecuadorian Amazon has been zoned into blocks, but not yet offered to multinational oil companies. Newer oil operations from the 1990s and this decade (Blocks 15, 16, and 31) have built new access roads into the primary forests of the Yasuní region. At the time of writing, Ecuador's Constituent Assembly just completed a new Constitution prohibiting extraction in protected areas except by Presidential petition in the name of national interest.

In Bolivia, two leased Amazonian exploration blocks cover ∼15,000 km^2^, including large parts of Madidi and Isiboro Securé National Parks and Pilon-Lajas Biosphere Reserve. Activity on these blocks has stalled for several years, but recent Bolivian newspaper reports indicate that exploration in this region is imminent [41]. Multinational oil companies operate these blocks, but now the state oil companies of Bolivia and Venezuela are joining forces to explore the region. In August 2007, Bolivian President Evo Morales and Venezuelan President Hugo Chavez created a new company composed of the state oil companies of the two nations [42]. One of the primary tasks of this new company is to explore for oil in the newly created blocks surrounding Madidi.

In 2005, the Brazilian government leased out 25 contiguous blocks surrounding the Urucu and Jurua gas fields in the state of Amazonas, bringing the total leased area to ∼67,000 km^2^. These new blocks lie within a largely intact part of the Brazilian Amazon [43]. The Urucu fields already contain producing gas wells, but the Jurua field, discovered in 1978, has yet to be exploited. A nearly 400 km roadless gas pipeline is being constructed to connect the Urucu gas fields to Manaus [44]. Another pipeline has been proposed to carry gas over 500 km to Porto Velho in the state of Rondônia. Brazil's National Petroleum Agency has also recently announced plans to look for oil and gas in the Amazonian state of Acre on the border with Peru and Bolivia [45].

In the Colombian Amazon, 35 exploration and production blocks (∼12,300 km^2^) are concentrated within and around Putumayo Department on the border with Ecuador. Production in Putumayo peaked years ago, but much of the oil in this region and beyond may be yet untapped or undiscovered [46]. Colombia's Hydrocarbon Agency recently announced a new 2008 bidding round, featuring nine new blocks in Putumayo. Over 90% of the Colombian Amazon is currently free from oil activities.

## Discussion

In sum, more than 180 oil and gas blocks now overlap the most species-rich part of the Amazon, including areas having the world's greatest known diversity of trees, insects, and amphibians. The threat to amphibians is of particular concern, not only because so much of their global diversity is concentrated in the western Amazon, but also because they are already the most threatened vertebrate taxa worldwide [5]. Many blocks also cover protected areas — such as national parks in Ecuador and Bolivia and a variety of lower-level protected areas in Peru — that were originally established for biodiversity protection.

Many of the oil and gas blocks are in remote areas and overlap indigenous territories, both titled lands and areas utilized by peoples in voluntary isolation. Moreover, the scope and magnitude of planned activity appears unprecedented. For example, of the 64 blocks now covering the Peruvian Amazon, all but eight have been created since 2004.

Oil and gas development in the western Amazon has already caused major environmental and social impacts. Given the increasing scope and magnitude of planned hydrocarbon activity, these problems are likely to intensify without improved policies.

It is to those policies that we now turn. We consider the impacts of roads, the requirement of free, prior and informed consent, the special needs of peoples living in voluntary isolation, the use of strategic environmental impact assessments, and the role of the international community. In each case, the policies adopted will have significant impacts one way or another on the region's biodiversity and the fate of its indigenous peoples. This is not an exhaustive list, but topics our experiences suggest are the most important.

### Roads

Roads are one of the strongest correlates of Amazonian deforestation [47]–[48]. New access roads cause considerable direct impacts — such as habitat fragmentation — and often trigger even greater indirect impacts, such as colonization [30], illegal logging [49], and unsustainable hunting [27]–[28]. Animals often targeted by local and indigenous hunters are involved in key ecological processes such as seed dispersal and seed predation [50]. The overhunting of large primates, for example, has the potential to change the composition and spatial distribution of western Amazon forests due to the loss of these important seed dispersers [51]. Even a rough extrapolation from the oil extraction in previous decades suggests that the planned wave of oil and gas activity may similarly fragment and degrade largely intact forests over huge areas in coming years and decades.

Two Amazonian modeling efforts indicate that deforestation is concentrated in the eastern and southern Brazilian Amazon — areas with high road density — but the western Amazon is largely intact due its remoteness and lack of roads [9], [43]. Oil and gas blocks, however, now fill much of these remote areas. A primary concern is that new oil and gas projects could bring a proliferation of new access routes throughout the western Amazon. Indeed, pending oil and gas projects are currently the primary threat to areas in eastern Ecuador (Blocks 31 and ITT), northern Peru (Blocks 39 and 67), Peru's Camisea region, Brazil's Urucu region, and Bolivia's Madidi region.

Oil access roads are a main catalyst of deforestation and associated impacts. A report from scientists working in Ecuador concluded that impacts along new access roads could not be adequately controlled or managed, particularly in regards to actions of the area's local or indigenous peoples [52]. The report, along with opposition by the Waorani indigenous people, pressured the Ecuadorian government, which banned Petrobras from building a road into Yasuní National Park in July 2005. The government forced the company to redesign the project without a major access road. As of this writing, Petrobras plans to use helicopters to transport all materials, supplies, equipment, and people to and from the well sites, with oil flowing out via a roadless pipeline. This decision by the Ecuadorian government might set an important precedent for policy: no new oil access roads through wilderness areas. A major roadless oil project in Ecuador's Block 10 was the region's first example that such development is possible [53], and Block 15 also features a roadless pipeline with canopy bridges. Elimination of new roads could significantly reduce the impacts of most projects.

### Free, Prior and Informed Consent

Governments claim the authority to manage natural resources located on or below indigenous peoples territories for the public interest, while indigenous peoples claim that their rights to property and territory allow them the right to free, prior and informed Consent (FPIC) regarding proposed extractive projects on their lands [54]–[55].

The key distinction lies between consultation and consent. International law — namely the 1989 International Labour Organization's Indigenous and Tribal Peoples Convention No. 169 — clearly mandates that indigenous peoples be consulted about development projects on their territories [56]. Indeed, national regulations in Ecuador and Peru, for example, mandate such consultation [57]–[58]. The question is, do indigenous peoples have the right to reject a project planned on their territory after being properly consulted? The latest international instruments indicate “yes”. The United Nations Declaration on the Rights of Indigenous Peoples — adopted by the General Assembly in 2007 — emphasizes FPIC prior to government approval of any project affecting indigenous lands or territories [59]. Also in 2007, the Inter-American Court on Human Rights issued a landmark ruling, *Case of the Saramaka People v. Suriname*, that the State must ensure the right of local peoples to give or withhold their consent in regard to development projects that may affect their territory [55].

A prerequisite for effective FPIC procedures is that indigenous peoples possess legal title to their traditional lands. The Inter-American Human Rights System has dealt extensively with this issue. In 1998, the Inter-American Commission found that it is a violation of the American Convention on Human Rights (Article 21, Right to Property) for a government to grant an extractive concession without the consent of the indigenous peoples of the area. The Inter-American Court subsequently ruled that this right to property requires the titling of their traditional territory [60]. Although many communities and nationalities have obtained such title, others still have not (or else the process is incomplete). Given that most of the oil blocks in question are in indigenous areas, the resolution of who controls the land and its sub-surface resources will greatly influence the development of the region.

### Indigenous Peoples in Voluntary Isolation

The situations in Ecuador and Peru highlight two of the major issues concerning hydrocarbons and indigenous peoples in voluntary isolation: a lack of understanding of the full extent of the territories of peoples in voluntary isolation and debate regarding “intangibilidad” — or untouchablility — of their known territories.

In Ecuador, the government created a Zona Intangible (Untouchable Zone) to protect the territory of its two known isolated groups from oil development in 1999 and delimited the 7,580-km^2^ zone via Presidential Decree in January 2007. However, testimonies from local indigenous Waorani indicate that signs of the Taromenane and Tagaeri are sometimes seen in areas that are covered by oil blocks, north of and outside the Zona Intangible. Moreover, the Taromenane speared to death an illegal logger outside the northern limit of the Zona Intangible in March 2008 [61], the clearest evidence to date that they range outside the demarcated zone.

In Peru, the Law for the Protection of Isolated Peoples in Voluntary Isolation (Law 28736) was passed in May of 2006, and implementing Regulations were issued by Presidential Decree in October 2007. The “untouchable” character of protective reserves for peoples in voluntary isolation may be broken for the exploitation of natural resources deemed by the state to be in the public interest, a loophole that allows extraction of oil and gas. Another major issue in Peru concerns hydrocarbon activities in areas formally proposed to be reserves for peoples in voluntary isolation. At least 15 blocks overlap such proposed reserves.

In May 2006, the Inter-American Commission on Human Rights granted precautionary measures in favor of the two known groups in voluntary isolation in the Ecuadorian Amazon, the Tagaeri and Taromenane, due to threats they face from oil activities and illegal logging. These measures call for the government to prohibit the entry of “third persons” — which would include oil companies — into their territory. In March 2007, the Inter-American Commission urged the Peruvian government, again through precautionary measures, to protect the indigenous peoples in voluntary isolation in the Madre de Dios region from threats posed by illegal logging. In 2007, indigenous organizations made three more requests to the Inter-American Commission for precautionary measures needed to stem the threats to uncontacted peoples posed by oil and gas projects in Peru.

### Strategic Environmental Assessments

Nations of the region require project-specific Environmental Impact Studies (EIS) prior to oil and gas exploration or exploitation projects. The oil companies contract the firms to conduct the studies, a system that clearly lacks independent analysis. Moreover, there are typically no comprehensive analyses of the long-term, cumulative, and synergistic impacts of multiple oil and gas projects across a wider region, generally referred to as a Strategic Environmental Assessment (SEA) [62].

In Peru, hydrocarbon blocks now overlap 20 protected areas. Thirteen of these protected areas preceded creation of the oil blocks and lack compatibility studies required by the Protected Areas Law [63]. An SEA could deal with these types of issues.

For example, in the Napo Moist Forest ecoregion of northern Peru, 28 blocks form a nearly continuous oil zone. There has been almost no regional planning, no analysis of the cumulative and long-term impacts, and no strategic planning for long-term protections of biodiversity and indigenous peoples. No national parks exist in the region, so there are no areas strictly off-limits to oil development. Indeed, the mass of oil blocks overlap two lower-level protected areas, several proposed protected areas, numerous titled indigenous territories, and a proposed Territorial Reserve to protect the indigenous peoples in voluntary isolation living in the core of the region. The development of proper SEAs would potentially reduce the negative impacts across the wider region of the western Amazon.

### Role of International Community

In 2006, over half of Ecuador's total oil production went to the United States, including nearly 90% of the heavy crude coming out of the controversial OCP pipeline [64]–[65]. Much of the oil feeding this pipeline comes from projects in sensitive areas, such as Yasuní National Park. In Peru, American, Canadian, European, and Chinese companies drive the exploration and exploitation of the Amazon.

Ecuador has proposed an innovative opportunity [66] for the world to share in the responsibility of protecting the Amazon. In April 2007, the President of Ecuador, Rafael Correa, announced that the government's preferred option for the largest untapped oil reserve, located beneath Ecuador's principal Amazonian national park (Yasuní), is to leave it permanently underground in exchange for compensation from the international community. The oil fields, known as Ishpingo-Tiputini-Tambococha (ITT), are within one of the most remote and intact parts of Yasuní National Park, and are part of the ancestral territory of the Waorani.

### Summary

While the history of oil and gas extraction in the western Amazon is one of massive ecological and social disruption, the future need not repeat the past. Roadless extraction would greatly reduce environmental and social impacts. Proper attention to the rights of indigenous peoples and the outright protection of lands of peoples living in voluntary isolation, who, by definition cannot give informed consent, would bring exploration within widely accepted international norms of social justice. Disinterested, regional scale strategic environmental assessments would prevent piecemeal damage across large areas. Finally, the international community can play a role in widening the options available to the region's nations and its indigenous peoples.

## Methods

Most data on oil blocks and pipelines are from government sources and were publicly available online at the time of submission. These include Colombia's Agencia Nacional de Hidrocarburos (http://www.anh.gov.co), Ecuador's Ministerio de Minas y Petróleos (http://www.menergia.gov.ec), Peru's Perupetro (http://www.perupetro.com.pe) and Ministerio de Energía y Minas (http://www.minem.gob.pe/hidrocarburos/index.asp), Bolivia's Ministerio de Hidrocarburos y Energía (http://www.hidrocarburos.gov.bo), and Brazil's Agência Nacional do Petróleo, Gás Natural e Biocombustíveis (http://www.anp.gov.br). When necessary, downloaded maps of boundaries of oil blocks and their attributes were digitized using ArcGIS 9.2.

We also collected information from major newspapers of the region, particularly El Comercio in Ecuador and La Razon in Bolivia.

Boundaries of protected areas are from the World Database of Protected Areas [67]. We digitized the boundaries of Parque Nacional Ichigkat Muja - Cordillera Del Condor, Santiago – Comaina, and Sierra del Divisor from maps available from the Instituto Nacional de Recursos Naturales (http://www.inrena.gob.pe). We divided protected areas into strictly (I to III) and less strictly (IV to VI) protected groups according to the IUCN categories for protected areas [68]. These categories range from I to VI, with lower numbers representing management to maintain natural ecosystems and processes, while higher numbers represent management oriented towards human recreation and sustainable resource extraction.

We converted biodiversity data for birds [69]–[70], mammals [71]–[72], and amphibians [73] to raster format and analyzed them in ArcGIS. For birds, we used only the breeding range for each species.

Size estimates of blocks were calculated using ArcGIS and verified by comparing to published accounts in government sources.

To calculate the percentage of Ecuadorian and Peruvian Amazon zoned into oil and gas blocks, we used the data in [74] for the size of the Ecuadorian Amazon (81,000 km^2^) and in [9] for the size of the Peruvian Amazon (677,048 km^2^). For the latter, see Table S2, Figure S2 from their Supplementary materials.

We analyzed indigenous territory maps in Peru [75] and Ecuador [R. Sierra, unpublished data] and recorded the number of overlaps with oil and gas blocks.

## Supporting Information

Abstract S1 (0.03 MB DOC)

Abstract S2 (0.03 MB DOC)
