# Supplementary material for: Oil and Gas Projects in the Western Amazon: Threats to Wilderness, Biodiversity, and Indigenous Peoples
Source: PLoS One. 2008 Aug 13;3(8):e2932. doi: 10.1371/journal.pone.0002932 (PMC2518521; doi:10.1371/journal.pone.0002932)
Supplement: Abstract S1 — (0.03 MB DOC) [file pone.0002932.s001.doc]

**Resumen**

*Antecedentes*

El occidente de la Amazonía es la porción biológicamente más rica de la Cuenca Amazónica y el hogar de una gran diversidad de grupos indígenas étnicos entre los que se hallan algunos de los últimos pueblos sin contacto que viven en aislamiento voluntario. En contraste con el este de la Amazonía Brasilera, el occidente de la Amazonía es un ecosistema que en gran parte se halla intacto. Bajo este paisaje se hallan reservas enormes de petróleo y gas, muchas aún sin explotar. La creciente demanda global está conduciendo a exploración y desarrollo sin antecedentes en la región.

*Metodología/ Hallazgos principales*

Sintetizamos información de fuentes gubernamentales para cuantificar la situación en cuanto a desarrollo petrolero en el occidente de la Amazonía. Los gobiernos nacionales limitan zonas geográficas específicas o “bloques” que son establecidos para actividades hidrocarburíferas y que pueden concesionarlas para exploración y producción a compañías estatales y multinacionales en el área de la energía. Cerca de 180 bloques, para explotación de petróleo o gas, cubren hoy aproximadamente 688.000 km^2^ en el occidente de la Amazonía. Estos bloques se sobreponen sobre la porción más rica en especies de la Amazonía. También hemos determinado que muchos de estos bloques se sobreponen sobre territorios indígenas que comprenden tierras con títulos y áreas utilizadas por pueblos en aislamiento voluntario. En el Ecuador y en el Perú, más de dos tercios de la Amazonía están cubiertos por bloques para explotación de petróleo o gas. En Bolivia y el occidente del Brasil, se pondrá en marcha el incremento rápido de actividades de exploración de gran importancia.

*Conclusiones/ Significado*

Si no se mejoran las políticas, el ámbito y magnitud creciente de la extracción planificada implican la probable intensificación de los impactos ambientales y sociales. Examinamos aquí los problemas más apremiantes en cuanto a políticas de conservación relacionadas con petróleo y gas que enfrenta la región. Éstos incluyen la necesidad de estudios de impacto ambiental estratégicos regionales y el uso de técnicas de extracción sin carreteras. También consideramos los conflictos donde los bloques se sobreponen sobre territorios de pueblos indígenas.
