# Supplementary material for: Oil and Gas Projects in the Western Amazon: Threats to Wilderness, Biodiversity, and Indigenous Peoples
Source: PLoS One. 2008 Aug 13;3(8):e2932. doi: 10.1371/journal.pone.0002932 (PMC2518521; doi:10.1371/journal.pone.0002932)
Supplement: Abstract S2 — (0.03 MB DOC) [file pone.0002932.s002.doc]

# Resumo

*Contexto*

A Amazônia ocidental é a região de maior riqueza biológica da bacia amazônica e abriga uma grande diversidade de etnias indígenas, incluindo alguns dos últimos grupos não-contactados do Planeta, vivendo em isolamento voluntário. Ao contrário da Amazônia oriental brasileira, a Amazônia ocidental é ainda um ecossistema na sua maioria intacto. Sob essa paisagem se encontram grandes reservas de petróleo e gás, muitas ainda não exploradas. A crescente demanda global está levando a uma exploração e desenvolvimento sem precedentes na região.

*Métodos/Principais Achados*

Sintetizamos informações de fontes governamentais para quantificar a situação do desenvolvimento petroleiro na Amazônia ocidental. Os governos nacionais delimitam zonas geográficas específicas ou “blocos” para exploração de hidrocarbonetos e podem arrendar esses blocos para exploração e produção a companhias de energia estatais ou multinacionais. Cerca de 180, blocos para exploração de petróleo ou gás, cobrem hoje aproximadamente 688.000 km^2^ da Amazônia ocidental. Esses blocos se sobrepõem com as áreas de maior riqueza de espécies da Amazônia. Determinamos também que muitos blocos se sobrepõem com terras indígenas, tanto terras homologadas como áreas utilizadas por povos em isolamento voluntário. No Equador e Peru, mais de dois terços da Amazônia estão cobertos por blocos para exploração de petróleo ou gás. Na Bolívia e no Brasil ocidental, atividades de exploração de grande porte devem crescer rapidamente.

*Conclusões/Importância*

Sem uma melhoria das políticas públicas, o crescente âmbito e magnitude da exploração planejada implicam numa provável intensificação dos impactos ambientais e sociais. Examinamos aqui as mais importantes políticas ambientais relacionadas com a exploração de petróleo e gás na região. Estas incluem a necessidade de estudos regionais estratégicos de impacto ambiental e a adoção de técnica de extração sem construção de estradas. Também consideramos os conflitos aonde blocos se sobrepõem com terras indígenas.
